# Computational Simulations Highlight the IL2Rα Binding Potential of Polyphenol Stilbenes from Fenugreek

**DOI:** 10.3390/molecules27041215

**Published:** 2022-02-11

**Authors:** Apoorva M. Kulkarni, Shraddha Parate, Gihwan Lee, Yongseong Kim, Tae Sung Jung, Keun Woo Lee, Min Woo Ha

**Affiliations:** 1 Department of Bio & Medical Big Data (BK4 Program), Division of Life Science, Research Institute of Natural Science (RINS), Gyeongsang National University (GNU), 501 Jinju-daero, Jinju 52828, Korea; apoorvamk19@gmail.com; 2 Division of Applied Life Science, Plant Molecular Biology and Biotechnology Research Center (PMBBRC), Gyeongsang National University (GNU), 501 Jinju-daero, Jinju 52828, Korea; parateshraddha@gmail.com (S.P.); pika0131@naver.com (G.L.); 3 School of Cosmetics and Food Development, Kyungnam University, Masan 631-701, Korea; kimys@kyungnam.ac.kr; 4 Laboratory of Aquatic Animal Diseases, Research Institute of Natural Science, College of Veterinary Medicine, Gyeongsang National University, 501-201, 501 Jinju-daero, Jinju-si 52828, Gyeongsangnam-do, Korea; jungts@gnu.ac.kr; 5 Jeju Research Institute of Pharmaceutical Sciences, College of Pharmacy, Jeju National University, 102 Jejudaehak-ro, Jeju 63243, Jeju-do, Korea; 6 Interdisciplinary Graduate Program in Advanced Convergence Technology & Science, Jeju National University, 102 Jejudaehak-ro, Jeju 63243, Jeju-do, Korea

**Keywords:** phytochemicals, stilbenes, IL2Rα, rhaponticin, fenugreek, drug discovery, molecular docking, molecular dynamics simulations, binding free energy, ADMET

## Abstract

Widely used in global households, fenugreek is well known for its culinary and medicinal uses. The various reported medicinal properties of fenugreek are by virtue of the different natural phytochemicals present in it. Regarded as a promising target, interleukin 2 receptor subunit alpha (IL2Rα) has been shown to influence immune responses. In the present research, using in silico techniques, we have demonstrated the potential IL2Rα binding properties of three polyphenol stilbenes (desoxyrhaponticin, rhaponticin, rhapontigenin) from fenugreek. As the first step, molecular docking was performed to assess the binding potential of the fenugreek phytochemicals with IL2Rα. All three phytochemicals demonstrated interactions with active site residues. To confirm the reliability of our molecular docking results, 100 ns molecular dynamics simulations studies were undertaken. As discerned by the RMSD and RMSF analyses, IL2Rα in complex with the desoxyrhaponticin, rhaponticin, and rhapontigenin indicated stability. The RMSD analysis of the phytochemicals alone also demonstrated no significant structural changes. Based on the stable molecular interactions and comparatively slightly better MM/PBSA binding free energy, rhaponticin seems promising. Additionally, ADMET analysis performed for the stilbenes indicated that all of them obey the ADMET rules. Our computational study thus supports further in vitro IL2Rα binding studies on these stilbenes, especially rhaponticin.

## 1. Introduction

Fenugreek (*Trigonella foenum*-*graecum* L.) belongs to the Fabaceae family and has been used as an important culinary ingredient in many households. Currently, 70 to 97 different species of fenugreek are being cultivated around the world [1]. Fenugreek contains a repertoire of phytochemicals and has been used for nutritional, nutraceutical, medicinal, and therapeutic purposes [2]. It has been used in the treatment of various disorders, such as cardiovascular diseases, hypercholesterolemia, hyperglycemia, cancer, liver ailments, and sexual disorders such as testosterone deficiency syndrome [1,3]. Major bioactive compounds present in fenugreek are believed to be polyphenol stilbenes, such as rhaponticin, and flavones such as isovitexin [4]. Researchers have also been successful in separating other stilbenes, such as desoxyrhaponticin and rhapontigenin, from the fenugreek extract [5]. The stilbenes desoxyrhaponticin, rhaponticin, and rhapontigenin have been predicted to have considerable health-promoting or pharmacological potential [6,7]. Stilbene is one of the most potent scaffolds in medicinal chemistry [8,9]. It is characterized by two aromatic rings linked by an ethylene moiety [10]. Plants produce stilbene to protect themselves against UV radiation, heat, insects, and microbial attacks [10]. Raloxifene, toremifene, or tamoxifen are some of the approved stilbene-based drugs. Several other stilbenes including resveratrol, tapinarof, and pterostilbene are undergoing clinical trials for chemoprevention-NCT04266353, cystic fibrosis-NCT04166396, chronic obstructive pulmonary disease- NCT03819517, plaque psoriasis-NCT03956355, NCT03983980, NCT04053387, endometrial carcinoma-NCT03671811, acute kidney injury-NCT04342975, etc. [10]. 

IL2Rα (interleukin 2 receptor subunit alpha) is a protein-coding gene, the product of which acts as a receptor for IL2. The collaboration of IL2 with its receptor, including IL2Rα, activates pathways that regulate cell survival and proliferation, such as the PI3K/AKT, RAS/RAF/MEK/ERK, and STAT5 pathways. Thus, the elevated expression of IL2Rα is witnessed in several cancers, such as leukemia, lymphoma, lung, breast, head-and-neck, and prostate [11]. Evidence from previous studies has shown IL2Rα overexpression to be related to adverse outcomes and poor therapy response, especially in elder AML (acute myeloid leukemia) patients [12,13]. While several small molecules have been reported for inhibition of IL2 [14,15], the research for IL2Rα-specific small molecules still needs to be explored [16].

**Figure 1:**
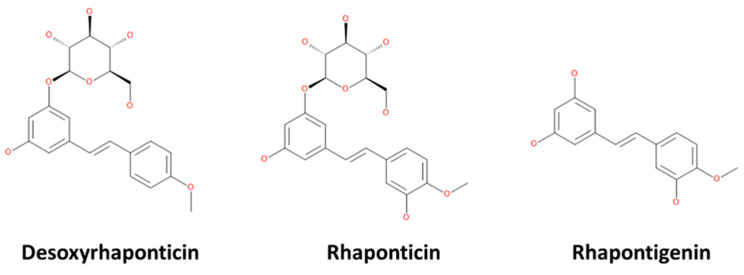
2D structure of the phytochemicals.

Computer-aided drug discovery (CADD) approaches have revolutionized the drug discovery process. Everything right from the identification of drug targets to the assessment of potential toxicity of the drugs can now be performed using in silico approaches. They offer the convenience of both cost and time effectiveness. To date, CADD has been successfully employed to bring new drug compounds to market for diverse diseases, including human immunodeficiency virus (HIV)-1-inhibiting drugs (atazanavir, saquinavir, indinavir, and ritonavir), anti-cancer drugs (raltitrexed), and antibiotics (norfloxacin) [17]. Our study thus focused on the computational exploration of potential IL2Rα-binding abilities of the phytochemicals, including desoxyrhaponticin, rhaponticin, and rhapontigenin from fenugreek. The 2D chemical structures of the phytochemicals researched in this study are shown in Figure 1.

## 2. Material and Methods

### 2.1. Receptor and Ligands Preparation

The protein structure of IL2Rα bearing PDB id: 3NFP (chain I) [18] was obtained from the Research Collaboratory for Structural Bioinformatics Protein Data Bank (RCSB PDB). Cleaning of the protein, i.e., removal of water and heteroatoms was performed with the Discovery Studio v18 (DS) [19] module, *Clean Protein*. Missing residues were added using the *Insert Loop* module of DS. Ligands, i.e., desoxyrhaponticin, rhaponticin, and rhapontigenin were retrieved from PubChem [20]. The minimization of the ligands was performed in DS with the *Minimize Ligands* module, before subjecting them to docking analysis.

### 2.2. Molecular Docking

Genetic Optimisation for Ligand Docking (GOLD) version 5.2.2 [21] was utilized to conduct molecular docking. Due to the unavailability of any small molecule bound IL2Rα structure in PDB, active site residues were determined based on the binding of the therapeutic antibody, daclizumab to IL2Rα, and this list was provided as input for GOLD docking. The selection of the best-docked pose was assessed on the criteria that the pose should possess a higher GoldScore fitness score and should demonstrate interactions with active site residues of the IL2Rα binding domain.

### 2.3. Molecular Dynamics (MD) Simulation

Molecular dynamics (MD) simulations were performed using GROningen Machine for Chemical Simulations (GROMACS) v5.0.6 package [22]. CHARMm27 was chosen as the desired force field for IL2Rα and topologies for stilbenes were generated using the SwissParam forcefield generation tool [23]. The simulations were carried out in a TIP3P water model containing a dodecahedron water box. Counter chloride ions were added to neutralize the charge of the system. The systems were then energy minimized using the Steepest Descent algorithm. A two-phase equilibration of the system was carried out: constant number N, volume V, and temperature T (NVT) and constant number N, pressure P, and temperature T (NPT) ensembles. NVT equilibration was performed at 300 K for 1 ns and NPT equilibration at 1 bar pressure for 1 ns with a Parrinello-Rahman barostat [24]. Following these equilibration steps, the simulations were carried out for 100 ns. 

### 2.4. Binding Free Energy

The free intermolecular binding energy between phytochemicals and IL2Rα was determined using molecular mechanics/Poisson–Boltzmann surface area (MM/PBSA) [25]. To employ these in GROMACS, the g_mmpbsa tool was utilized [26]. The binding free energy of a protein-ligand complex (Δ*G_bind_*) in solution is specified as:

$$\Delta G_{bind}=G_{complex}-\left[G_{protein}+G_{ligand}\right]$$

Here, *G_complex_* implies the sum of the free energy of the protein-ligand complex, and *G_protein_* and *G_ligand_* imply the free energies of protein and ligand in their unbound states. 

*G_solv_* i.e., the solvation term is the combination of polar, *G_polar,_* and nonpolar contribution, *G_nonpolar_*:

$$\Delta G_{solv}=\Delta G_{polar}+\Delta G_{nonpolar}$$

*G_nonpolar_* i.e., the non-polar contribution is proportional to the solvent accessible surface area (SASA):

$$\Delta G_{nonpolar}=\gamma \left(SASA\right)+\beta$$

where *γ* = 0.0227 kJ mol^−1^ Å^−2^ and *β* = 3.849 kJ mol^−1^.

### 2.5. Physiochemical Properties and ADMET Prediction

The assessment of physiochemical properties and ADMET prediction was performed in SwissADME [27] and pkCSM [28].

## 3. Results and Discussion

### 3.1. Receptor Preparation

**Figure 2:**
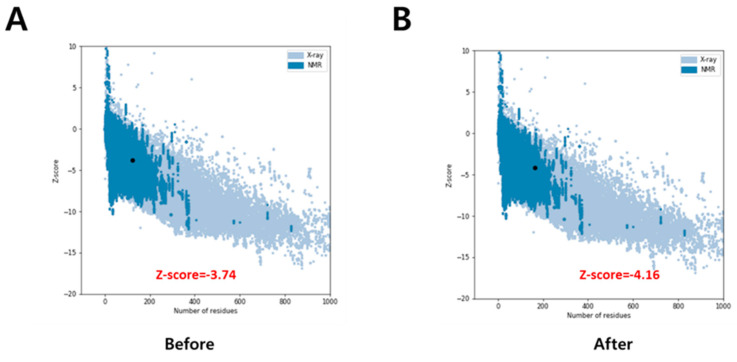
ProSA Plot for protein structure evaluation. (**A**) Before (**B**) After modeling of gap residues.

The *Clean Protein* module of DS identified two gaps in the chosen IL2Rα structure. One of them was between Lys31 and Arg36 and the other one between Ser64 and Pro101. Based on the IL2Rα sequence obtained from Uniprot ID: P01589, the sequence to fill these gaps was determined as RGFR and ATRNTTKQVTPQPEEQKERKTTEMQSPMQPVDQASL. Insert Loop was then utilized to model the gaps using the identified sequences. Further, the protein structure was accessed in ProSA before and after the gap substitution. ProSA is frequently employed in structure validations and calculates the Z-score, which indicates the overall quality of the structure. For an acceptable quality structure, the Z-score should be within a range characteristic of native proteins. The ProSA plot along with the Z-score is shown in Figure 2. Based on the Z-score of −4.16, it was inferred that the overall quality of the protein was maintained after the modeling of missing residues, and this structure was employed in subsequent studies.

### 3.2. Molecular Docking Analysis

**Figure 3:**
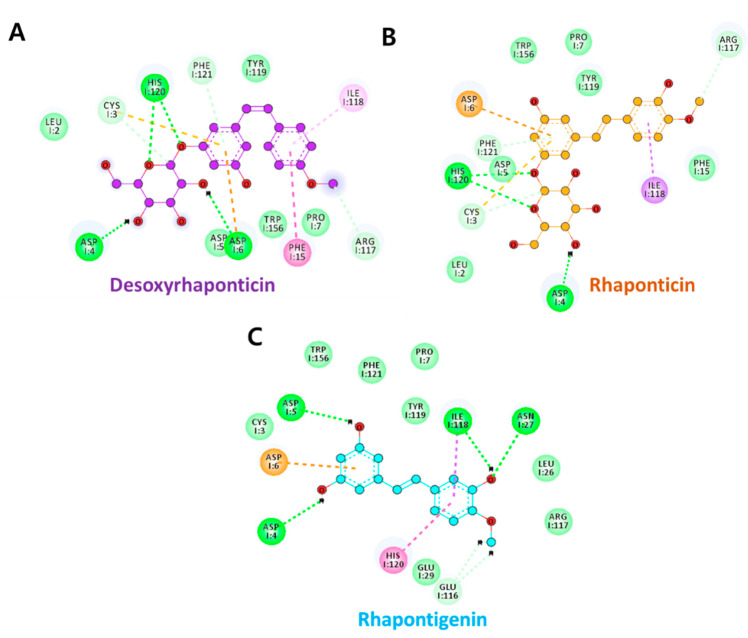
Molecular docking interactions of (**A**) Desoxyrhaponticin, (**B**) Rhaponticin, and (**C**) Rhapontigenin with IL2Rα. Green dashed lines indicate hydrogen bonds. All the other dashed lines represent various types of π hydrophobic bonds. Light green colored spheres specify the residues participating in van der Waals interactions.

The aim of molecular docking is to predict the best binding mode of a ligand to a macromolecular partner [29], and structures of both ligand and the receptor are a prerequisite. There are no crystal structures available for IL2Rα in complex with small molecule inhibitors and, to the best of our knowledge, our research is the first to exploit small molecule-based computational-assisted drug discovery for IL2Rα. As daclizumab had demonstrated high affinity and high specificity for IL2Rα, and owing to the availability of IL2Rα-daclizumab crystal structure, we utilized IL2Rα from PDB id: 3NFP as our receptor and binding region of daclizumab to IL2Rα ectodomain, as the active site. From the binding analysis of daclizumab with IL2Rα, it was learned that a total of 12 hydrogen bonds are established with residues Leu2, Asp4, Asp6, Asn27, Tyr43, His120, Gly152, Thr154, and Arg155. Additionally, two salt bridges are formed, one each with Asp4 and Asp6. Since hydrogen bonds and salt bridges form a central role in protein binding, the residues involved in these bonds were given as the GOLD input list of active site residues. GoldScore fitness comprises of hydrogen bond and van der Waals energy of protein ligand, ligand internal van der Waals energy, and ligand torsional strain energy. The GoldScore fitness of the best-docked pose of desoxyrhaponticin, rhaponticin, and rhapontigenin with IL2Rα was 58.73, 58.58, and 45.47, respectively. All three ligands demonstrated hydrogen bonds with His120. Desoxyrhaponticin demonstrated additional hydrogen bonds with Asp4 and Asp6. Rhaponticin and rhapontigenin also demonstrated hydrogen bonds with Asp4 and Asp5, respectively. These, along with the complete list of other interactions, are shown in Figure 3.

### 3.3. MD Simulation Analysis

Although molecular docking is popularly used to detect binding poses of ligands with macromolecular proteins, most of the docking programs treat protein as rigid molecules, and fail to consider the conformational changes during the ligand binding events [30], hence resulting in a possibility of detecting an inaccurate ligand binding profile. To address this issue, molecular dynamics is frequently employed post molecular docking. Molecular dynamics is a computational technique that stimulates the flexible conduct of the molecular system as a function of time [29]. Correspondingly, molecular dynamics simulations were conducted for four molecular systems i.e., (A) IL2Rα-desoxyrhaponticin, (B) IL2Rα-rhaponticin, and (C) IL2Rα-rhapontigenin and (D) IL2Rα alone. 

#### 3.3.1. Root Mean Square Deviation (RMSD) and Root Mean Square Fluctuation (RMSF)

**Figure 4:**
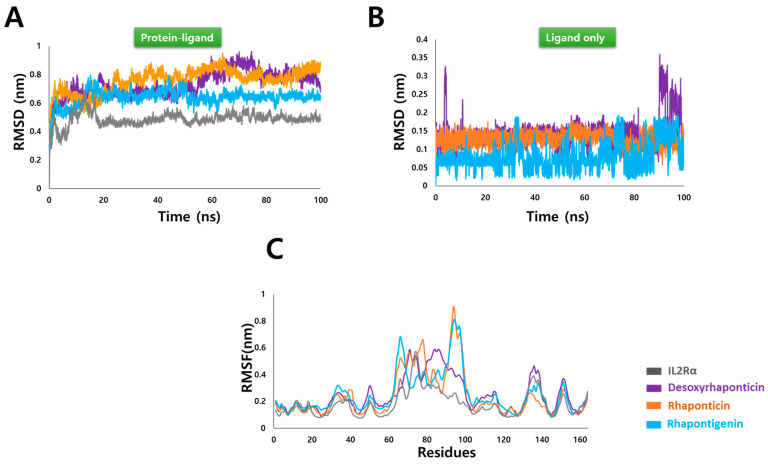
Stability analysis after molecular dynamics (MD) simulations. (**A**) RMSD of IL2Rα-phytochemical complexes (**B**) RMSD of ligands alone, and (**C**) RMSF of IL2Rα-phytochemical complexes.

RMSD measures the deviation of the atomic position and indicates any structural changes throughout the MD simulation. RMSD analysis was performed for the protein-ligand complexes and ligands only. Figure 4A illustrates the stable overall RMSD for IL2Rα-phytochemical complexes. The average RMSD obtained for IL2Rα-desoxyrhaponticin, IL2Rα-rhaponticin, and IL2Rα-rhapontigenin was 0.73 ± 0.08 nm, 0.76 ± 0.08 nm and 0.63 ± 0.05 nm, respectively. Although these were higher than the average protein RMSD i.e., 0.44 ± 0.04 nm, the visual analysis of the RMSD throughout the trajectory, as shown in Figure 4A, confirms the stability of the systems. To determine if the conformation of phytochemicals had undergone any changes during MD, RMSD for ligands only were determined. The ligand-only RMSD profiles are depicted in Figure 4B. Rhaponticin showed a very stable profile with an average RMSD of 0.12 ± 0.01 nm. Desoxyrhaponticin showed some fluctuations during the initial and last 10 ns but maintained an average of 0.14 ± 0.04 nm. The average RMSD of rhapontigenin was calculated as 0.07 ± 0.03 nm. Thus, RMSD observations indicated the stability of ligands alone as well as in complex with Il2Rα.

RMSF is useful in accessing the per-residue fluctuation in a protein molecule as a consequence of ligand binding. As is depicted in Figure 4B, other than residues 60–100, no significant fluctuations were observed in all the systems. Residue 60–100 corresponds to the previously modeled segment in IL2Rα and happens to be a loop. Loop fluctuations are a common observation in MD and this segment does not belong to the chosen binding site, therefore the fluctuations observed in this region were considered insignificant for the present study. Overall, the RMSF analysis also pointed towards the stability of the IL2Rα-ligand complexes.

#### 3.3.2. Hydrogen Bond Analysis

**Figure 5:**
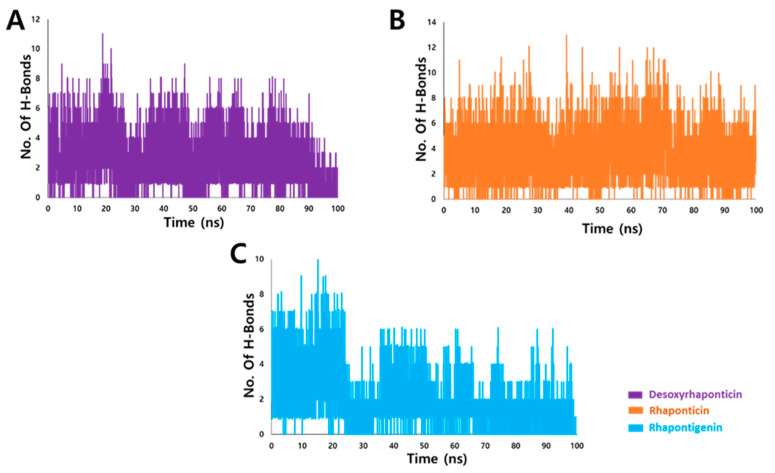
Hydrogen bond analysis after molecular dynamics (MD) simulations of IL2Rα with (**A**) Desoxyrhaponticin (**B**) Rhaponticin and (**C**) Rhapontigenin.

Generally, hydrogen bonds are considered as promoters of protein-ligand interaction [31,32,33], and therefore, analysis of hydrogen bonds is an important consideration post MD. Rhaponticin with an average number of 3.6 bonds formed the highest number of hydrogen bonds with IL2Rα throughout the 100 ns simulation. Desoxyrhaponticin and rhapontigenin formed an average of 2.6 and 2.0 hydrogen bonds. The number of hydrogen bonds of IL2Rα-ligand complexes during the 100 ns simulation is shown in Figure 5.

**Figure 6:**
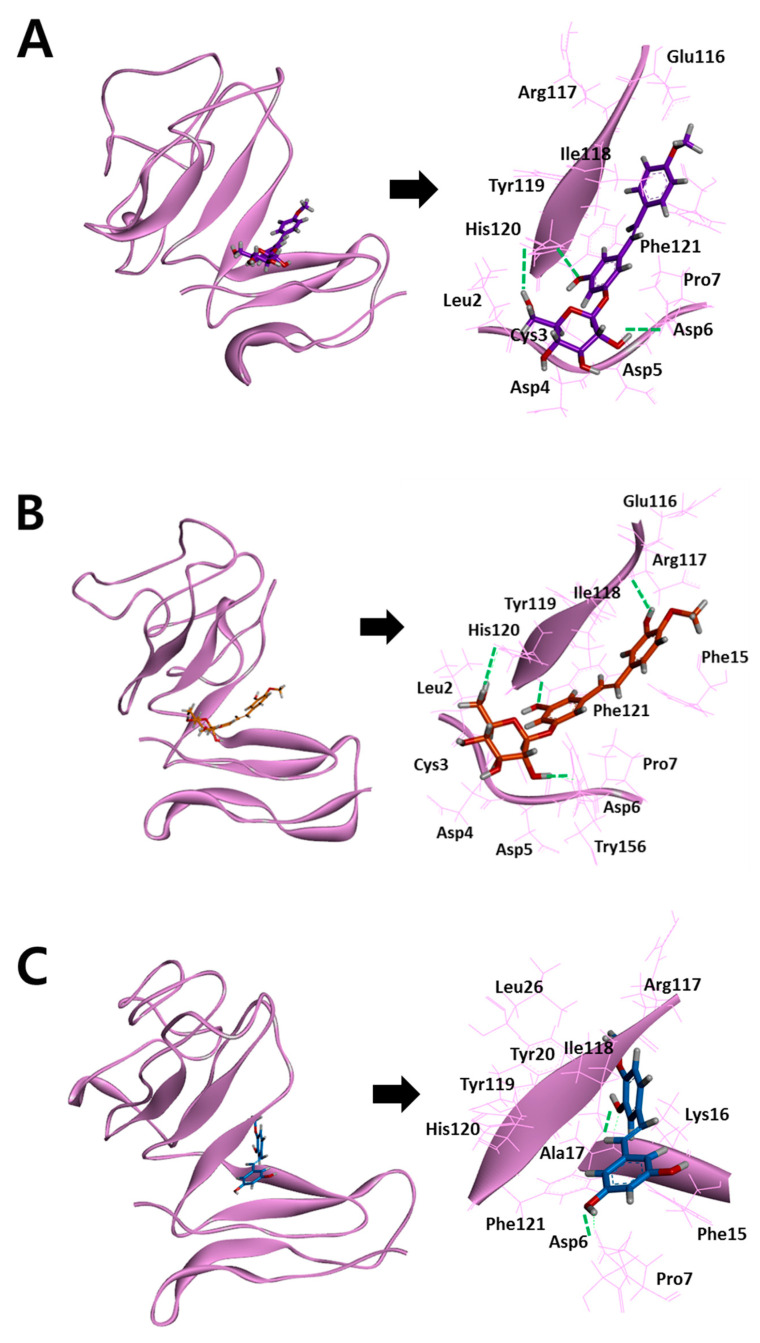
Interactions of IL2Rα-phytochemical complexes: (**A**) Desoxyrhaponticin (**B**) Rhaponticin (**C**) Rhapontigenin. Hydrogen bonds are indicated in green dashed lines. Other residues are either involved in π or hydrophobic bonds, details of which are presented in Table 1.

**Figure 7:**
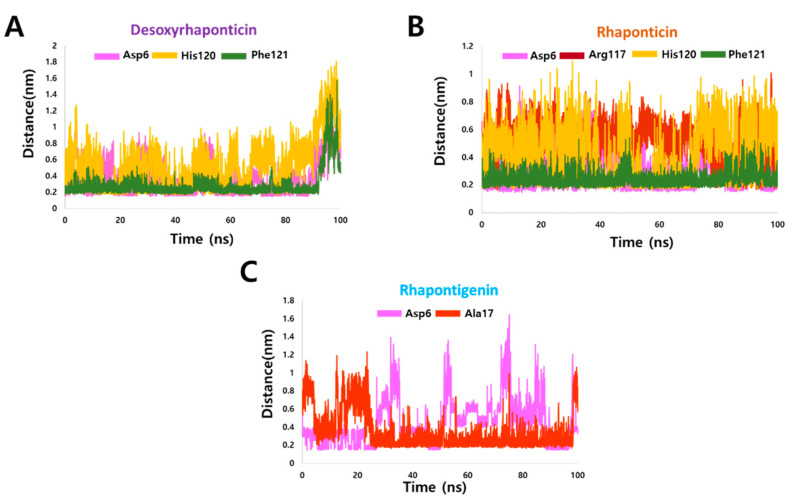
Distance profiles of Il2Rα residues involved in a hydrogen bond with (**A**) Desoxyrhaponticin (**B**) Rhaponticin (**C**) Rhapontigenin during the MD simulation.

Next, it was important to evaluate whether active site residues were involved in hydrogen bonding with the ligands under consideration. For this purpose, we initially clustered all the frames post MD using the gmx cluster, and the least RMSD frame was selected as a reference pose from the largest cluster. Using DS, the interactions of the reference pose were delineated. Figure 6 shows all the ligand-binding amino acids of IL2Rα with green dotted lines representing the hydrogen bonds. All three phytochemicals demonstrated hydrogen bond interaction with Asp6. Desoxyrhaponticin demonstrated additional hydrogen bonds with His120 and Phe121. Rhaponticin formed three additional hydrogen bonds with Arg117, His120, and Phe121. Rhapontigenin formed one additional hydrogen bond with Ala17. For energetically significant and stable hydrogen bonds, usually, a distance cut off of ≤0.35 nm for hydrogen bond-forming atoms is employed [34]. Therefore, to access the stability of the hydrogen bonds formed, we aimed at knowing the distance between hydrogen bond-forming atoms of protein and the ligands throughout the 100 ns run. The hydrogen bond distance profiles are provided in Figure 7. The average distances between the atoms involved in the hydrogen bond formation are indicated in Table 1. For desoxyrhaponticin, it was observed that the hydrogen bond with Phe121: HN was the most reliable with an average distance of 0.28 nm. The average distance between H39 and Asp6:OD2 was 0.37 nm. However, the average distance between the hydrogen bond-forming atoms of Phe120 and desoxyrhaponticin was 0.52, thus signifying the unreliability of this bond. For rhaponticin too, with an average distance of 0.24 nm, the hydrogen bond between Phe121: HN and O7 was most reliable. With Asp6:OD1, H40 of rhaponticin maintained an average distance of 0.31 nm. The average distances between the hydrogen bond-forming atoms of rhaponticin and Arg117 and His120 were more than 0.35 nm, implying the probable insignificance of these bonds. Rhapontigenin’s O2 with Ala17: HN maintained an average distance of 0.35 nm. However, the average distance between Asp6:OD1 and rhapontigenin H33 was below 0.35 nm during the first 26 ns after which the average distance started increasing. The distance analysis indicates that rhaponticin is most likely to form a greater number of reliable hydrogen bonds compared to desoxyrhaponticin and rhapontigenin.

From the interaction analysis of IL2 with IL2Rα, it was learned that residues 1–6, 25–29, 35–43, 57, 64, and 118–120 of IL2Rα interact with IL2 [18]. Apart from these hydrogen bonds, several electrostatic and hydrophobic interactions were also observed and are outlined in Table 1. Generally, protein-protein interactions are critically dependent on few ‘hot spot’ residues of the interaction surface and these residues might disrupt the interaction if mutated. The analysis of IL2-IL2Rα interaction (PDB id: 1Z92) [35] analyzed from a protein-protein interaction hotspot predictor-HSPred [36], showed that residues, Leu2, Cys3, Asp4, Asp5, Asp6, Ile118, and His120 were among the hotspot residues (Data not shown). Thus, the phytochemicals (especially rhaponticin) are able to engage with essential IL2Rα residues and might further aid in the disruption of IL2-IL2Rα interaction.

### 3.4. Binding Free Energy Analysis

**Figure 8:**
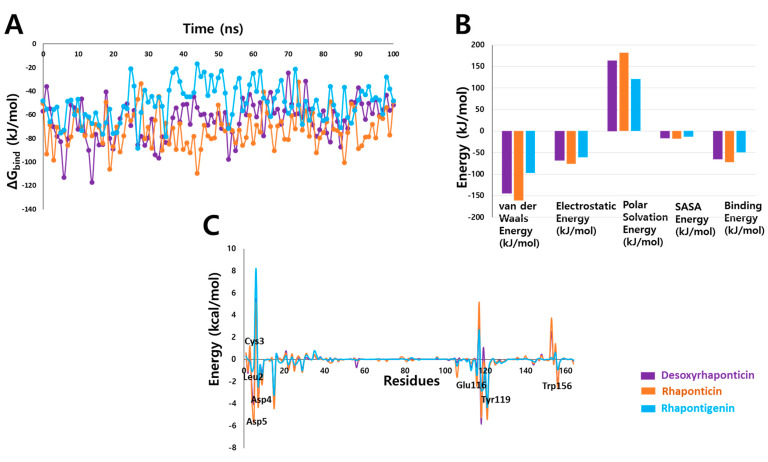
(**A**) Binding free energies of IL2Rα-phytochemicals (**B**) Decomposition of binding free energies and (**C**) Per-residue contribution to binding free energy.

The binding affinity is the change in the free energy associated with a binding process. The magnitude of this affinity can be an estimate of how strongly a ligand might interact with its protein [37]. Therefore, this is an important step in the computational drug discovery process. With an average binding free energy of −72.477 ± 14.598 kJ/mol (Figure 8A), rhaponticin might be better at IL2Rα binding than desoxyrhaponticin and rhapontigenin, whose average binding free energies are −64.768 ± 16.680 kJ/mol and −49.487 ± 15.326 kJ/mol, respectively. It is important to mention here that the error estimations in the binding free energy calculations are dependent on several factors, including the execution of a single long simulation rather than multiple small runs, the number of frames selected, and the inclusion of the entire trajectory for analysis. From the decomposition analysis of the binding free energies (Figure 8B), it was learned that van der Waals energy was the significant factor. In agreement with this, the per-residue energy contribution highlighted in Figure 8C shows that residues Leu2, Asp4, Asp5, Glu116, Tyr119, and Trp156 have been among the major contributors, especially for rhaponticin. On the other side, although a van der Waals interaction was established with Cys3 (Table 1), positive energy values were observed for this residue. Upon further scrutiny, it was learned that in IL2Rα, Cys3 is involved in intramolecular disulfide bond with Cys147 [18], and therefore, the interaction of Cys3 with the phytochemical might not be of great significance. Additionally, residues Pro7 and Phe15, which were involved in π-interactions, have also contributed favorably.

### 3.5. Physicochemical Properties and ADMET Analysis

To access the absorption, distribution, metabolism, excretion, and toxicity of investigational drugs, ADMET is frequently employed. Assessment of these parameters helps in drug optimization and avoids the late-stage failures amounting to a considerable loss of time and resources [38]. Table 2 lists the ADMET and physicochemical properties of the phytochemicals under study. The Caco-2 monolayer of the cell is widely used to predict the oral absorption of cells. Desoxyrhaponticin and rhaponticin have demonstrated low Caco-2 permeability. Human intestinal absorption was also measured where the results predicted that a little over 50% of desoxyrhaponticin and rhaponticin would be through the human intestine, where ~92% of rhapontigenin was predicted to be absorbed by the intestine. Distribution studies highlight that all three phytochemicals are not blood-brain barrier or central nervous system penetrators. Since cytochrome P450s can regulate the metabolism of various drugs, inhibition of isoforms of the CYP450 superfamily, namely CYP1A2, CYP2C19, CYP2C9, CYP2D6, and CYP3A4 was checked. Except for rhapontigenin, which showed inhibition of CYP1A2, CYP2C9, our phytochemicals of interest do not show CYP isoform inhibition. Excretion primarily includes hepatic and renal clearance. It is related to bioavailability and is important for determining dosing rates to achieve steady-state concentrations [28]. Using the total clearance predictor of pkCSM, the excretion values ranged from 0.08 to 0.13 mL/min/kg. Drug-induced liver injuries and allergic contact dermatitis are major toxicity concerns presented during the drug development process [28]. None of the tested phytochemicals present hepatotoxicity or skin sensitization. Additionally, the blockade of hERG K(+) channels may potentially lead to fatal arrhythmia and hERG inhibitors are recognized as a primary anti-target in safety pharmacology [39]. None of the phytochemicals were predicted to be hERG inhibitors. Toxicity studies thus indicate that all the tested phytochemicals are potentially nontoxic. Drug-likeness is another important consideration during drug discovery protocols. To access drug-likeness, Lipinski’s and Veber’s rules were accessed in the SwissADME server. To qualify Lipinski’s parameters of a drug, the candidate compound should have a number of hydrogen bond acceptors ≤ 10, hydrogen bond donors ≤ 5, molecular weight < 500 Da, and LogP (the logarithm of octanol-water partition coefficient) ≤ 5 [40]. Veber’s rule states that if a drug has 10 or fewer rotatable bonds and polar surface area equal to or less than 140 Å^2^, then the probability of good oral bioavailability would be high [41]. Although desoxyrhaponticin and rhapontigenin satisfy all these criteria, rhaponticin shows a higher TPSA than the standard and might possibly exhibit bioavailability issues. To tackle these issues, usage of PEGylated liposomes containing rhaponticin [42] and synthesis of a folate receptor-targeted rhaponticin conjugate [43] have been proposed in previous studies.

## 4. Conclusions

Increased expression of IL2Rα leading to increased interaction with its host IL2 causes the activation of several pathways. Therefore, IL2Rα is a promising clinical target. Epidemiological studies have shown that diet can influence several cancers. For example, a higher intake of fruits and vegetables has been shown to lower the risk of colorectal cancer [44]. The research undertaken in the present investigation aimed at unraveling the IL2Rα binding potential of the stilbenes from fenugreek, namely, desoxyrhaponticin, rhaponticin, and rhapontigenin using a combination of molecular docking and molecular dynamics simulations. Desoxyrhaponticin and rhaponticin showed comparable molecular docking scores. However, rhaponticin interacted with a large number of IL2Rα residues and displayed higher binding energy while maintaining stability when bound to IL2Rα. Our research thus indicates that compared to desoxyrhaponticin and rhapontigenin, rhaponticin might probably be a more effective IL2Rα inhibitor. It is also important to highlight here that targeting protein-protein interactions with small molecule inhibitors has always been considered challenging. However, in the past couple of decades, several researches have shown that small molecules can act as effective protein-protein inhibitors and more than 40 such molecules are currently in pre-clinical development [45]. Owing to these, the present research thus provides computational proof of the binding of desoxyrhaponticin, rhaponticin, and rhapontigenin to IL2Rα and necessitates the need for in vitro validation, especially with rhaponticin.

## Figures and Tables

**Table 1:** Interaction details of IL2Rα-phytochemical complexes.

Compound	Hydrogen Bonds	Avg. Distance (nm)	π-Interactions	van der Waals Interactions
Desoxyrhaponticin	Asp6:OD2-H39	0.37	Asp5, Pro7, Phe15, Ile118	Leu2, Cys3, Glu116, Arg117, Tyr119
	His120:ND1-H40	0.52		
	Phe121:HN-O7	0.28		
Rhaponticin	Asp6:OD1-H40	0.31	Pro7, Phe15, Ile118	Leu2, Cys3, Asp4, Asp5, Glu116, Tyr119, Trp156,
	Arg117:O-H51	0.51		
	His120:ND1-H41	0.42		
	Phe121:HN-O7	0.24		
Rhapontigenin	Asp6:OD1-H33	0.44	Phe15, Ile118, Tyr119	Leu26, Arg117, His120, Phe121
	Ala17:HN-O2	0.35		

**Table 2:** ADMET and physicochemical properties of the phytochemicals under investigation.

	Parameters	Standard	Desoxyrhaponticin	Rhaponticin	Rhapontigenin
**Properties**	Molecular Weight (Da)	≤500	404.41	420.41	258.27
	*LogP*	≤5	0.74	0.45	2.98
	Rotatable bonds	≤10	6	6	3
	H-bond acceptors	≤10	8	9	4
	H-bond donors	≤5	5	6	3
	TPSA (Å^2^)	≤140	128.84	149.07	69.92
**Absorption**	Human intestinal absorption (%)	>30%: perfectly absorbed	58.29	50.00	91.20
	Permeability Caco-2 cell (Log Papp in 10^−6^ cm/s)	>0.90: well absorbed	−0.15	−0.07	0.86
**Distribution**	BBB permeability	<−1: poorly distribute to the brain	−1.10	−1.25	−0.82
	CNS permeability	<−3: unable to penetrate CNS	−3.63	−3.80	−2.22
**Metabolism**	Cytochrome P450 inhibitors	-	No	No	Yes (CYP1A2, CYP2C9)
**Excretion**	Total clearance	-	0.13	0.06	0.08
**Toxicity**	AMES	-	No	No	No
	hERG I/II inhibitor	-	No	No	No
	Hepatotoxicity	-	No	No	No
	Skin Sensitization	-	No	No	No

## Data Availability

Data are contained within the article.
